# Editorial: Exosomes, miRNAs, and lncRNAs in breast cancer: Therapeutic and diagnostic applications

**DOI:** 10.3389/fgene.2023.1192866

**Published:** 2023-05-09

**Authors:** Zahra Sadat Hashemi, Mahlegha Ghavami, Manoj Kumar Kashyap

**Affiliations:** ^1^ Breast Cancer Research Center, Motamed Cancer Institute, Tehran, Iran; ^2^ Department of Pathology, Dalhousie University, Halifax, NS, Canada; ^3^ Amity Stem Cell Institute, Amity Medical School, Amity University Haryana, Gurugram, Haryana, India; ^4^ Clinical Biosamples and Research Services (CBRS), Noida, Uttar Pradesh, India

**Keywords:** breast cancer, lncRNA, microRNA, circRNA, exosome, triple-negative breast cancer

Breast cancer is a complex disease that can be influenced by a variety of factors, including genetics, lifestyle, and environmental factors. It is classified into different subtypes based on the presence or absence of hormone receptors and HER2 expression, as well as the signature profile of the gene of the tumor cells. The presence of estrogen and progesterone receptors, which are proteins that respond to female hormones, is a paramount factor not only in breast cancer development but also treatment.

Tumors that express these receptors are referred to as ER-positive or PR-positive, and can often be treated with hormone therapy. HER2 is another protein that plays a role in the growth and division of cells, and tumors that overexpress HER2 are referred to as HER2-positive. In addition to these receptor-based subtypes, breast cancer can also be classified based on gene expression profiles, which can provide insight into the underlying biology of the tumor and help guide treatment decisions. Some common gene expression subtypes include HER2-enriched, luminal A, and B type, and triple-negative (when ER, PR, and HER2 are absent) breast cancer.

Exosomes are small extracellular vesicles (EVs) that are released by cells and are decisive in intercellular communication. Exosomes possess biomolecules like DNA/RNA, lipids, or proteins. Recent research has shown that exosomal circRNAs (circular RNAs) can play a role in breast cancer progression by regulating gene expression and signaling pathways. CircRNAs are a type of non-coding RNA that forms a covalently closed continuous loop, which makes them more stable and resistant to degradation compared to linear RNAs. Circular RNAs (circRNAs) are a type of RNA molecule that have been found to play important roles in regulating gene expression. They are formed by covalently linking the 3′and 5′ends of a linear RNA molecule, resulting in a circular structure. One of the ways circRNAs can regulate gene expression is by acting as miRNA sponges. miRNAs are small non-coding RNAs that can bind to specific sequences in messenger RNAs (mRNAs), leading to their degradation or repression of translation. CircRNAs can contain miRNA-binding sites that compete with mRNAs for miRNA binding, effectively sequestering the miRNAs and preventing them from interacting with their target mRNAs. This can lead to increased expression of the target mRNAs, and thus altered gene expression. CircRNAs can also regulate gene expression by interacting with RNA-binding proteins (RBPs), which are molecules that bind to specific RNA sequences and can affect RNA stability, splicing, and translation. CircRNAs can act as sequestering molecules for RBPs, binding to them and preventing them from interacting with other RNA molecules. This can lead to altered gene expression, as the RBPs may normally play a role in regulating the stability, splicing, or translation of specific mRNAs. In addition to these mechanisms, circRNAs can also interact with other RNAs or proteins, leading to altered gene expression. For example, some circRNAs have been found to interact with transcription factors, chromatin-modifying enzymes, or other regulatory proteins, leading to changes in gene expression. Overall, circRNAs are a diverse and complex class of RNA molecules that can play important roles in regulating gene expression in a variety of ways. Exosomal circRNAs can be used as biomarkers for breast cancer diagnosis and prognosis, as their expression levels have been shown to correlate with tumor size, lymph node metastasis, and overall survival. Additionally, targeting exosomal circRNAs may represent a novel therapeutic strategy for breast cancer treatment.

MicroRNAs regulate gene expression via binding to mRNAs and preventing their translation into proteins. Dysregulation of microRNAs has been implicated in cancer development and progression. LncRNAs are a class of RNA molecules >200 nucleotides in length that do not encode proteins and are shown to be decisive in gene regulation and cellular processes, and their aberrant regulation is linked with different cancers. CircRNAs form closed-loop structures by covalent means. CircRNAs is decisive in the regulation of gene, as well as in cellular processes in conditions like cancer. Overall, the study of exosomes and their cargo, including microRNAs, lncRNAs, and circRNAs, is an active area of research in cancer biology, with the potential to uncover new insights into cancer development and progression and to identify druggable targets for anti-cancer therapy.

The study by Chen et al. highlights the significance of autophagy, tumor immune microenvironment, and long non-coding RNAs in breast cancer. The identification of 10 autophagy-related lncRNAs and their use in constructing a prognostic model through univariate and multivariate Cox analysis is a novel approach to predicting the prognosis of breast cancer. The negative correlation between the risk score and the prognosis of BRCA indicates that patients with high-risk scores may have a poor prognosis. The ARlncRNAs model has been proven to be a reliable independent prognostic factor for breast cancer. Gene Set Enrichment Analysis (GSEA) enrichment analysis suggests that patients with high-risk scores have several tumor-related pathways enriched, while those with low-risk scores have several immune-related pathways enriched. This finding indicates that patients with low-risk scores may have a better immune response to breast cancer. Patients in the low-risk group have higher immune scores and are more active in immune cells and immune pathways, which suggests that these patients may respond better to immunotherapy. Additionally, patients in the low-risk group have higher PD-1 and CTLA-4 expression levels, which make them more likely to benefit from Immune checkpoint inhibitors (ICIs) treatment. Overall, the ARlncRNAs model provides a useful tool for predicting the prognosis of breast cancer and guiding individualized treatment for patients. By identifying patients with low-risk scores, the model may help to select patients who are more likely to benefit from ICIs treatment, improving patient outcomes and quality of life (Chen et al.).

Inflammation is a normal response of the immune system to injury, infection, or tissue damage. It is a complex process that involves the activation of various immune cells, the release of inflammatory mediators, and the recruitment of immune cells to the site of inflammation. Inflammation is necessary for tissue repair and regeneration, but it can also contribute to the pathogenesis of many diseases, including cancer. Inflammation can have both pro-tumorigenic and anti-tumorigenic effects depending on the type and activation state of immune cells in the tumor microenvironment. CD8+/Th1 cells, NK cells, and M1 tumor-associated macrophages (TAMs) are associated with an anti-tumorigenic response, as they promote tumor destruction and inhibit tumor growth. On the other hand, CD4+/Th2 cells and M2 TAMs are associated with a pro-tumorigenic response, as they favor tumor progression by promoting angiogenesis, tissue remodeling, and immunosuppression. The balance between these different immune cell types and their activation states is critical in determining the outcome of the immune response and the progression of breast cancer. Inflammation can stimulate the production of reactive oxygen species (ROS), which can have both pro-tumorigenic and anti-tumorigenic effects depending on the signaling pathways involved. ROS can activate signaling pathways that promote cellular proliferation, survival, and migration, which can contribute to tumor growth and metastasis. On the other hand, ROS can also activate signaling pathways that promote apoptosis, senescence, and DNA repair, which can inhibit tumor growth. MiRNAs can be regulated by ROS and can, in turn, regulate the expression of genes involved in oxidative stress and inflammation. For example, miRNAs can regulate the expression of antioxidant enzymes and transcription factors that modulate ROS levels, and they can also regulate the expression of cytokines and chemokines that modulate the immune response. Inflammation is a key driver of breast cancer development and progression. Inflammatory cells, such as macrophages and neutrophils, produce ROS and cytokines that can promote tumor growth and metastasis. Inflammatory cytokines can also modulate miRNA expression, suggesting a potential link between miRNAs, ROS, and inflammation in breast cancer. Several miRNAs have been identified as regulators of ROS production and inflammation in breast cancer. For example, miR-146a has been shown to inhibit ROS production and inflammation by targeting the nuclear factor-κB (NF-κB) pathway. MiR-155 has been shown to promote inflammation and ROS production by targeting the suppressor of the cytokine signaling 1 (SOCS1) pathway. MiR-21 has been shown to promote ROS production and inflammation by targeting the antioxidant enzyme superoxide dismutase 2 (SOD2) pathway. Targeting miRNAs that regulate ROS production and inflammation may provide new opportunities for therapy in breast cancer. For example, miRNA mimics or inhibitors could be used to modulate the expression of specific miRNAs and thereby regulate ROS production and inflammation in breast cancer cells. By modulating the expression of these miRNAs, it may be possible to alter the evenness between pro-tumorigenic and anti-tumorigenic immune cells in the tumor microenvironment and modulate ROS production, which could have therapeutic benefits for breast cancer patients. In conclusion, the interplay between inflammation, ROS, and miRNAs is a complex process that plays a paramount role in breast cancer progression. A better understanding of this process may lead to the development of new therapeutic strategies for breast cancer patients, and ongoing research in this area is important for improving breast cancer treatment and patient outcomes (Villarreal-García et al.).

Copy number variations (CNVs) are alterations in DNA copies of a particular segment with correlated with the development and progression of cancer. Enhancers are DNA regions that regulate the expression of genes, and the presence of CNVs in these regions can affect gene expression levels. By integrating expression data, copy number data, and H3K27ac data (a histone modification associated with active enhancers), the researchers were able to identify CNA-driven enhancer-gene and enhancer-lncRNA pairs in each of the four breast cancer subtypes (Basal-like, Her2, LumA, and LumB). Furthermore, the study reconstructed a CNV-driven enhancer-lncRNA-mRNA regulatory network and identified potential prognostic biomarkers in different subtypes, including MUM1 and AC016876.1 as prognostic biomarkers in LumA and Basal-like subtypes, respectively, and enhancer-related lncRNA-mRNA pairs as prognostic biomarkers in different subtypes. The study’s findings suggest that targeting genes regulated by CNA-driven enhancers and enhancer-related lncRNA-mRNA pairs may have therapeutic potential in breast cancer treatment. The identification of prognostic biomarkers could also help clinicians better predict patient outcomes and tailor treatment plans accordingly (Zhao et al.).

Exosomal circRNAs are circular RNAs that are released from cells and found in exosomes. These exosomal circRNAs can either promote or suppress breast cancer by affecting various biological pathways. Recent studies have shown that exosomal circRNAs can influence breast cancer development and progression by regulating genes related to cell proliferation, invasion, and metastasis. Additionally, exosomal circRNAs have been shown to be involved in therapeutic resistance, making them an important target for the development of new cancer therapies. Although the exact mechanisms of exosomal circRNAs in breast cancer are not yet fully understood, researchers are continuing to explore the potential of these molecules as diagnostic and therapeutic targets. CircRNAs have shown promise as biomarkers for breast cancer diagnosis and prognosis, and they may also be used to monitor treatment response and detect early relapse. In summary, exosomal circRNAs play an important role in breast cancer development and progression, and they may have the potential as diagnostic and therapeutic targets. Future research will continue to investigate the underlying mechanisms of exosomal circRNAs in breast cancer and explore their clinical implications (Hussen et al.).

Breast cancer is a complex disease with various subtypes and diverse etiologies. Early diagnosis and optimal management are essential to reduce the mortality associated with breast cancer. Nanotechnology-based approaches, such as using exosomes as therapeutic tools, have emerged as potential strategies for the diagnosis and treatment of breast cancer. Exosomes are small, membrane-bound nanovesicles that are secreted by cells and contain a variety of biomolecules, including proteins, lipids, and nucleic acids such as miRNAs. Exosomal miRNAs are known to regulate gene expression and play a decisive role in cancer pathogenesis, making them attractive targets for the development of new diagnostic and therapeutic tools. Recent studies have shown that exosomal miRNAs have the potential as biomarkers for breast cancer diagnosis and prognosis. Exosomal miRNAs can be detected in various body fluids, including blood, urine, and breast milk, making them a non-invasive and easily accessible diagnostic tool. In addition, exosomal miRNAs can be used as therapeutic agents for breast cancer. They can be delivered to cancer cells via exosomes, thereby modulating gene expression and altering the cellular phenotype. This approach has the potential to overcome drug resistance and reduce off-target effects associated with conventional chemotherapy. In conclusion, exosomal miRNAs have emerged as promising tools for the diagnosis and treatment of breast cancer. Future research will continue to explore the role of exosomal miRNAs in breast cancer pathogenesis and their potential clinical applications (Singh et al.).

